# Recreating or Rcr3ating? Bioengineering homologs of the Rcr3 protease to activate plant immunity

**DOI:** 10.1093/plcell/koae187

**Published:** 2024-06-27

**Authors:** Bradley Laflamme

**Affiliations:** Assistant Features Editor, The Plant Cell, American Society of Plant Biologists; Department of Molecular Genetics, University of Toronto, Toronto, ON, Canada, M5S 1A1

Plant immunity hinges on the host's ability to recognize microbial invaders and initiate an appropriate defense response. Phytopathologists have collectively developed a strong understanding of the many ways that plants can achieve this recognition through their repertoire of immune receptors (Jones et al. 2024). Armed with this information, we have more opportunities than ever before to translate our knowledge of the molecular basis of plant immunity into potent, tuneable, and durable disease resistance for the crop species that feed the planet.

In this issue, **Jiorgos Kourelis and colleagues (Kourelis et al. 2024)** exploit our understanding of one tomato immune receptor, Cf-2, to engineer novel immune outputs. Cf-2 activates immunity by sensing pathogen-induced modifications to another host protein, the apoplastic protease Rcr3. Specifically, Cf-2 perceives the interaction between Rcr3 and Avr2, an effector protein secreted by the pathogenic fungus *Cladosporium fulvum* (Rooney et al. 2005). Indirect recognition of pathogen effectors through host “decoy” proteins such as Rcr3 is a common mechanism of plant immune activation, and previous work has shown that indirect immunity can be engineered de novo by changing the substrate specificity of decoy proteins to capture new effectors (Kim et al. 2016). In the case of the Cf-2-Rcr3-Avr2 module, we know that only a few of the tested Rcr3 homologs from solanaceous plants can play the role of decoy, since this requires both making a complex with Avr2 and being able to activate Cf-2 (Kourelis et al. 2020). And while we know a bit about the residues fulfilling these functions in tomato Rcr3, there is still much to learn about the protein features governing each of these roles if we are to exploit this mechanism for engineering immune outputs. Thus, in this work, Kourelis and colleagues took a collection of Rcr3 homologs and set about turning them into effective decoys.

The group began with the eggplant (*Solanum melongena*) ortholog of Rcr3 (*Sm*Rcr3), which can be inhibited by Avr2 but does not activate Cf-2 immunity. To identify regions of the protein responsible for this discrepancy, the group made several chimeric Rcr3 proteins by introducing fragments of the tomato (*Solanum lycopersicum*) Rcr3 into the *Sm*Rcr3 sequence. Then, using localized cell death as a marker for immune activation in *Nicotiana benthamiana*, the group screened these chimeras to identify portions of the tomato Rcr3 sequence responsible for Cf-2 activation. This led to the identification of a single proline residue that, when swapped into the *Sm*Rcr3 sequence, was sufficient to partially trigger Avr2/Cf-2-dependent cell death.

In contrast to *Sm*Rcr3, *N. benthamiana* Rcr3 (*Nb*Rcr3) is neither inhibited by Avr2 nor able to activate Cf-2 immunity (Kourelis et al. 2020). The team noted that *Nb*Rcr3 not only lacked a key residue (N194; G194 in *Nb*Rcr3) important for Avr2 binding (Kourelis et al. 2020) but also was completely missing 3 residues in the protease domain that are highly conserved in the Cf-2-activating Rcr3 homologs. A modified *Nb*Rcr3 protein with N194 and those 3 missing residues was able to robustly elicit Avr2/Cf-2–dependent cell death—but neither modification in isolation was sufficient. Indeed, the group highlighted that each of these modifications had a distinct role in making *Nb*Rcr3 an effective decoy: N194 enables inhibition by Avr2, while the 3 additional residues are required for activating Cf-2. These data elegantly highlight that decoy design requires an appreciation for all the key interactions that govern immune activation.

Finally, the group looked at tomato Pip1, a divergent paralog of Rcr3. While Avr2 can inhibit Pip1, this interaction does not trigger Cf-2–dependent cell death (Kourelis et al. 2020). Taking a similar approach to *Sm*Rcr3, the group generated and tested over 50 chimeric proteases based on Rcr3 and Pip1, 8 of which were both inhibited by Avr2 and able to trigger Cf-2–dependent cell death. Further probing into each of these Rcr3 fragments through another set of mutagenesis experiments identified several residues that were additively required for robust cell death. This information helped to generate Pip1+, a Pip1 protein containing 16 cell death–associated residues from Rcr3 that functions with similar efficacy to Rcr3 in triggering Cf-2 immunity (Figure). Thus, at 3 distinct levels of divergence from tomato Rcr3, a limited number of protein sequence changes can repurpose proteases to become immune-activating decoys.

**Figure.**
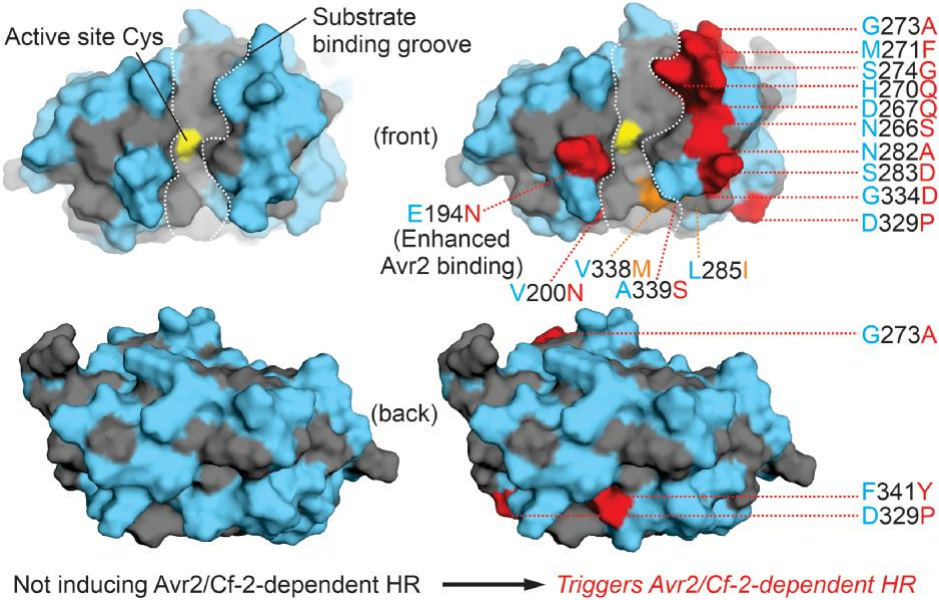
AlphaFold2 models for Pip1 (left) and Pip1+ (right). Residues conserved between Rcr3 and Pip1 are highlighted in blue, while residues specific to Pip1 are highlighted in grey. On the right (Pip1+), residues modified to create Pip1+ are highlighted in red, while 2 additional residues modified in Pip1++ (not discussed in this In Brief) are highlighted in orange. The active site cysteine is highlighted in yellow for both models. Reprinted from Kourelis et al. 2024 (Figure 5C).

Alongside their mutagenesis work, the group makes use of the protein structural prediction and modeling tools available through AlphaFold2 to highlight how their mutations are likely affecting protein activity. For instance, most of the modified residues that contribute to Cf-2 activation in Pip1+ are located in an uncharacterized β-sheet lobe, away from the binding pocket that is targeted by Avr2 (Figure), suggesting that this region of the protein may be responsible for physically interacting with Cf-2. Collectively, this study not only provides a tangible path toward bioengineering new immune co-receptors, but it will also help guide future investigations into the protein-protein interactions that are central to activating the Cf-2 receptor. It will be particularly exciting if we can further interrogate the interaction between Cf-2 and Rcr3 (a physical interaction is likely but has never been demonstrated), as this could open the doors toward developing new protease-receptor pairs in immunity to guard against other protease-targeting pathogens.
